# Correction: Lepore Signorile et al. c-MYC Protein Stability Is Sustained by MAPKs in Colorectal Cancer. *Cancers* 2022, *14*, 4840

**DOI:** 10.3390/cancers16213704

**Published:** 2024-11-01

**Authors:** Martina Lepore Signorile, Valentina Grossi, Candida Fasano, Giovanna Forte, Vittoria Disciglio, Paola Sanese, Katia De Marco, Francesca La Rocca, Raffaele Armentano, Anna Maria Valentini, Gianluigi Giannelli, Cristiano Simone

**Affiliations:** 1 Medical Genetics, National Institute of Gastroenterology Saverio de Bellis, IRCCS Research Hospital, Castellana Grotte, 70013 Bari, Italy; 2 Department of Pathology, National Institute of Gastroenterology Saverio de Bellis, IRCCS Research Hospital, Castellana Grotte, 70013 Bari, Italy; 3 Scientific Direction, National Institute of Gastroenterology Saverio de Bellis, IRCCS Research Hospital, Castellana Grotte, 70013 Bari, Italy; 4 Medical Genetics, Department of Biomedical Sciences and Human Oncology (DIMO), University of Bari Aldo Moro, 70124 Bari, Italy

## Error in Figure

In the original publication [1], there was a mistake in Figure 4f as published. One colony well of Figure 4f was erroneously assembled during the preparation of the figures. This oversight was due to an inadvertent cut and paste of the same colony well. The corrected Figure 4f appears below. The authors apologize for any inconvenience caused and state that the scientific conclusions are unaffected. This correction was approved by the Academic Editor. The original publication has also been updated.

## Figures and Tables

**Figure 4:**
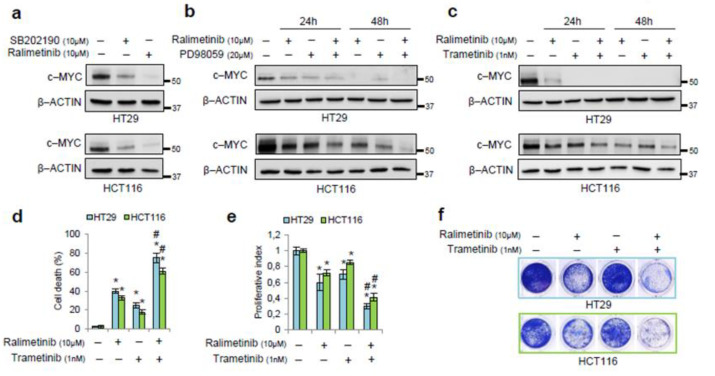

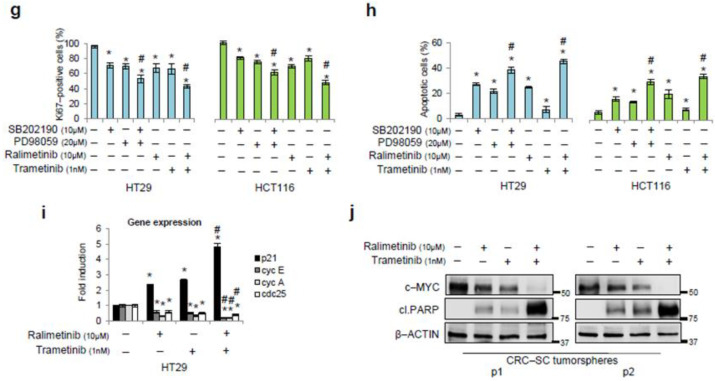
Pharmacological inhibition of p38α and MEK as a synthetic lethality approach. (**a**) Immunoblotting showing c-MYC protein levels in HT29 and HCT116 cells treated for 24 h with SB202190 (10 μM) or ralimetinib (10 μM). (**b**) Immunoblotting showing c-MYC protein levels in HT29 and HCT116 cells treated for up to 48 h with ralimetinib (10 μM) and/or PD98059 (20 μM). (**c**) Immunoblotting showing c-MYC protein levels in HT29 and HCT116 cells treated for up to 48 h with ralimetinib (10 μM) and/or trametinib (1 nM). (**d**) Quantification of cell death by trypan blue staining in HT29 and HCT116 cells treated with ralimetinib (10 μM) and/or trametinib (1 nM) for 48 h. (**e**) Proliferative index of HT29 and HCT116 cells treated with ralimetinib (10 μM) and/or trametinib (1 nM) for 48 h, as determined by WST-1 assay. (**f**) Cell viability assay on HT29 and HCT116 cells treated with ralimetinib (10 μM) and/or trametinib (1 nM) for 48 h. (**g**) Graphs summarizing the percentage of Ki67-positive cells, as determined by flow cytometry analysis, in HT29 and HCT116 cells treated with SB202190 (10 μM) and/or PD98059 (20 μM) or with ralimetinib (10 μM) and/or trametinib (1 nM) for 48 h. (**h**) Graphs summarizing the percentage of apoptotic cells (early + late), as determined by flow cytometry analysis of annexin V staining, in HT29 and HCT116 cells treated as in g. (**i**) RT-PCR analysis of the mRNA levels of the c-MYC target genes p21, Cyclin E, Cyclin A, and cdc25 in HT29 cells treated with ralimetinib (10 μM) and/or trametinib (1 nM) for 24 h. (**j**) Immunoblotting showing c-MYC and cleaved PARP protein levels in two lines of patient-derived CRC-SCs grown as tumorspheres (#9 and #40) treated with ralimetinib (10 μM) and/or trametinib (1 nM) for 48 h. (**a**–**c**,**j**) β-actin was used for normalization. (**d**,**e**,**g**–**i**) Statistical analysis was performed using Student’s *t*-test; * *p* < 0.05: vs. untreated cells, # *p* < 0.05: vs. corresponding single treatment; cl.PARP: cleaved PARP; p1: patient 1-derived CRC-SC; p2: patient 2-derived CRC-SC. The presented results are representative of at least three independent experiments. Detailed information about Western Blot can be found at supplementary materials.
