# Supplementary material for: c-MYC Protein Stability Is Sustained by MAPKs in Colorectal Cancer
Source: Cancers (Basel). 2022 Oct 4;14(19):4840. doi: 10.3390/cancers14194840 (PMC9562641; doi:10.3390/cancers14194840)

Supplementary Figure S1

a

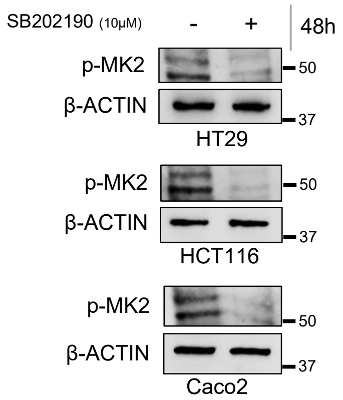

b

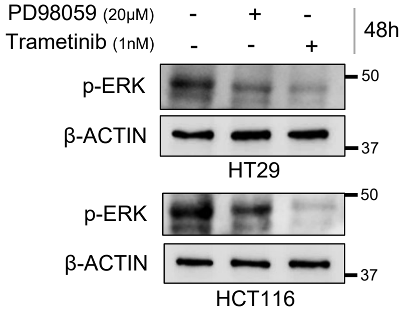

Supplementary Figure S2

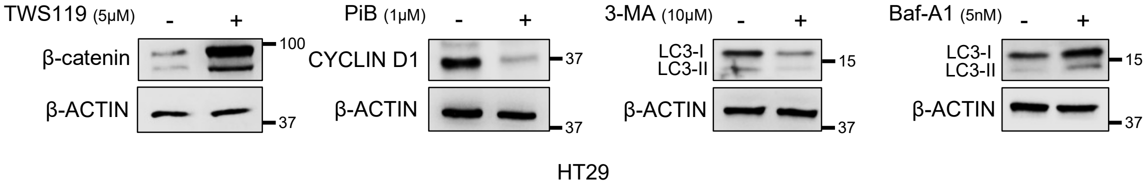

Supplementary Figure S3

a

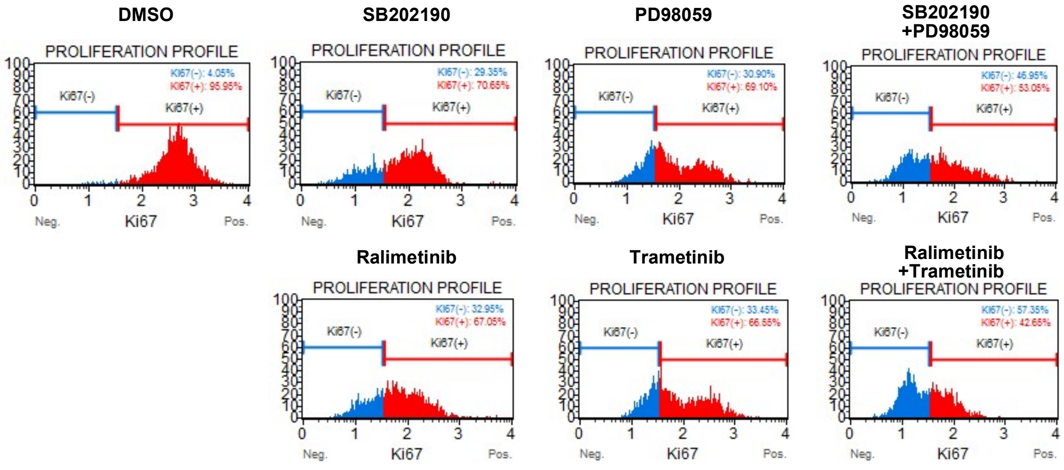

HT29

b

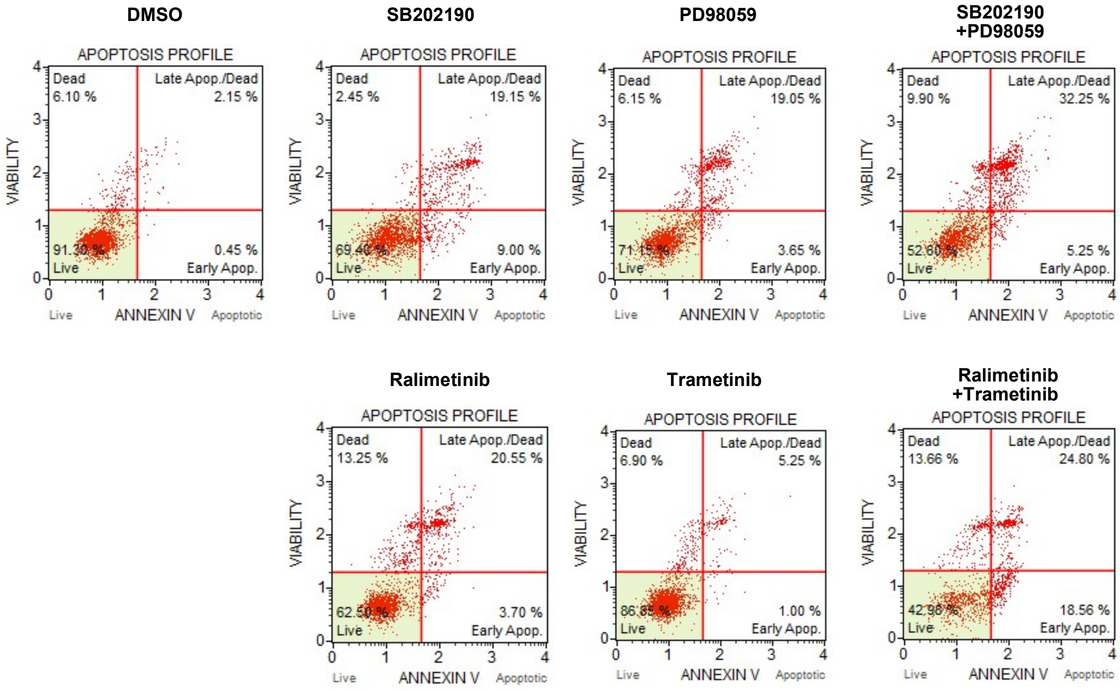

HT29

Figure 1 Original blots

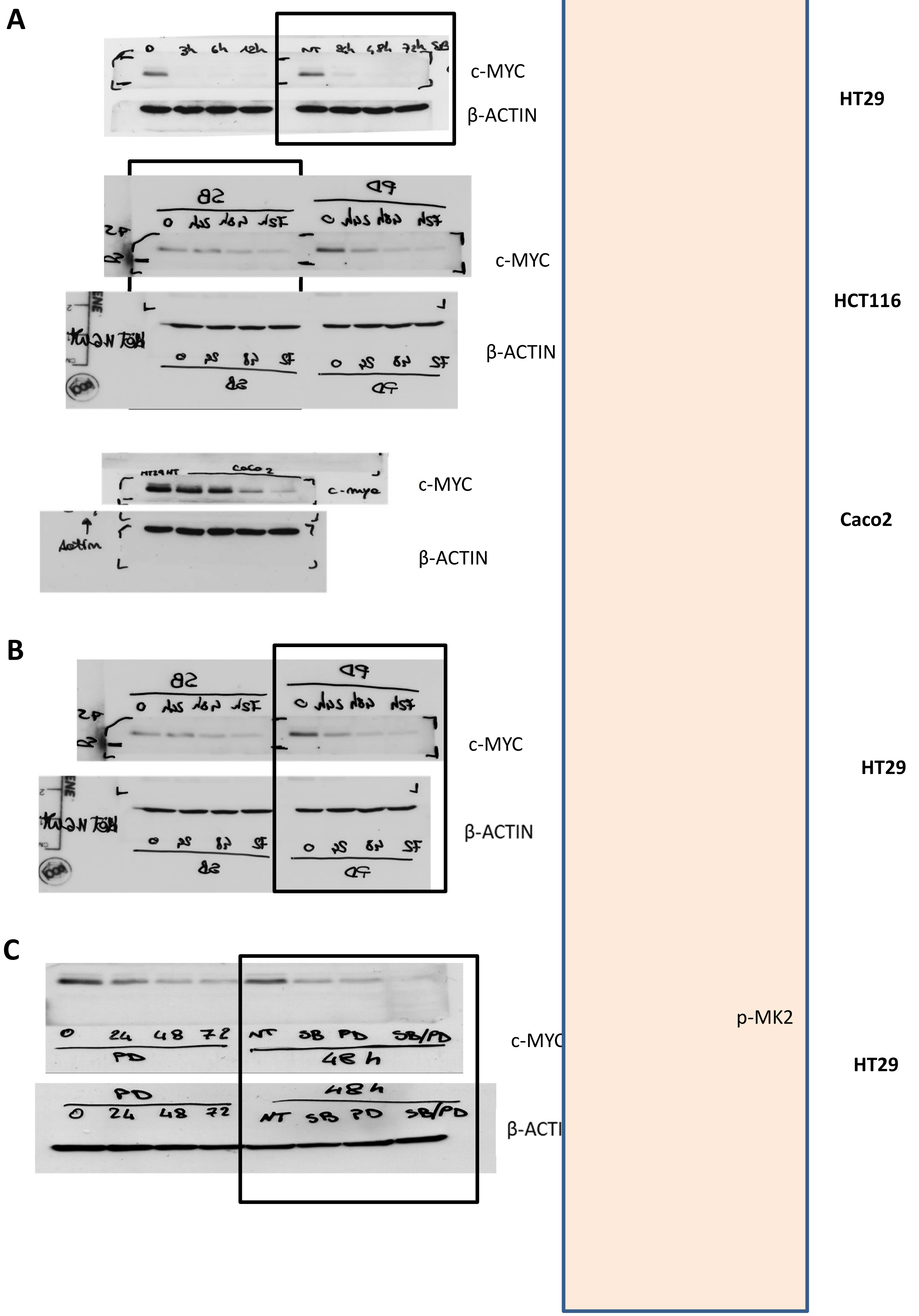

Figure 1 Original blots

Results obtained during revision process

D

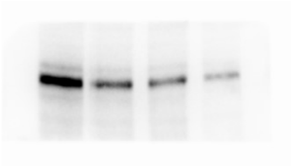

c-MYC

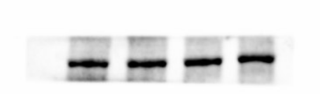

β-ACTIN

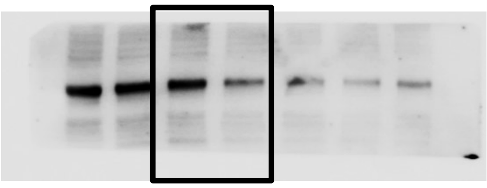

MEK

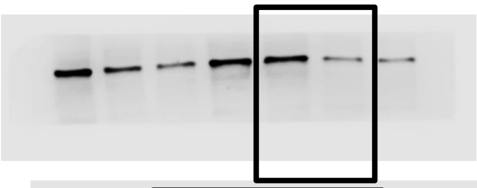

p38a

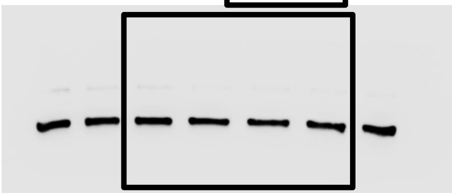

β-ACTIN

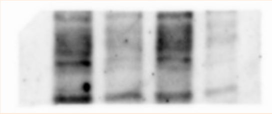

p-MK2

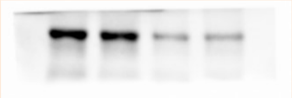

p-MEK1

Figure 2 Original blots

A

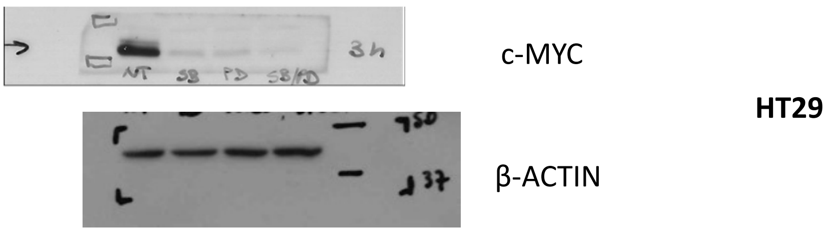

B

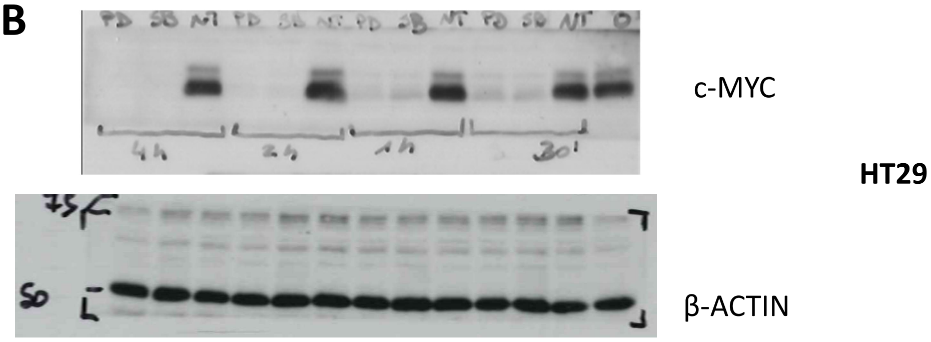

C

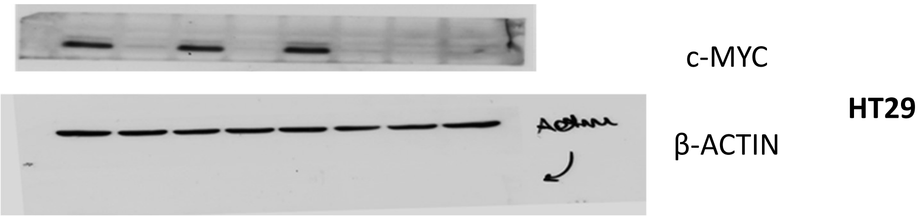

D

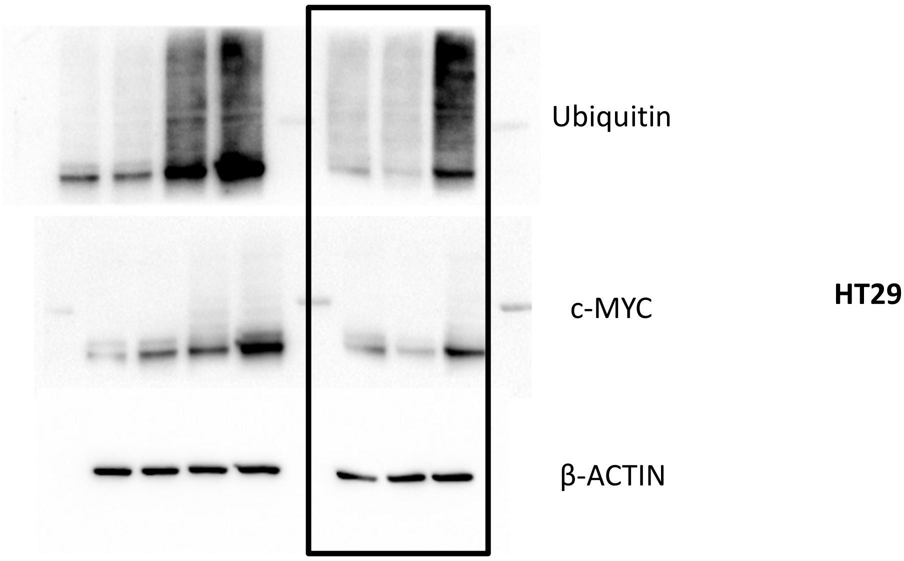

Figure 3 Original blots

A

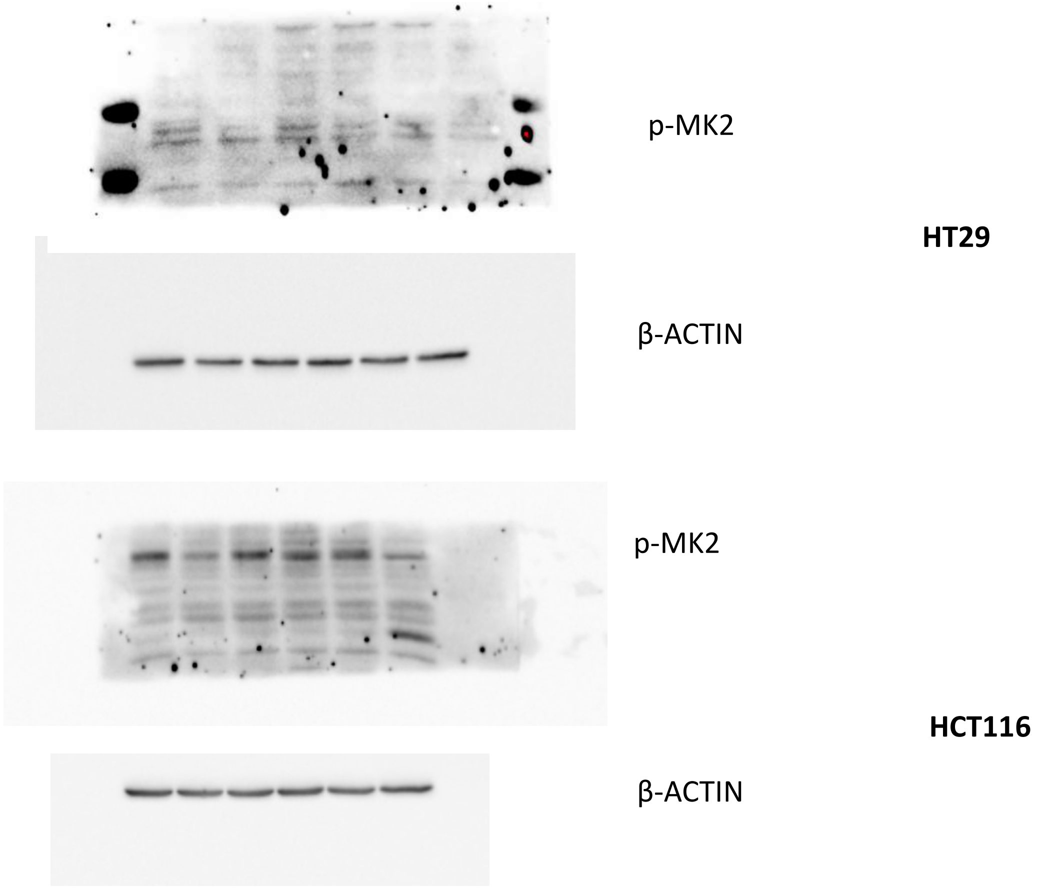

Figure 4 Original blots

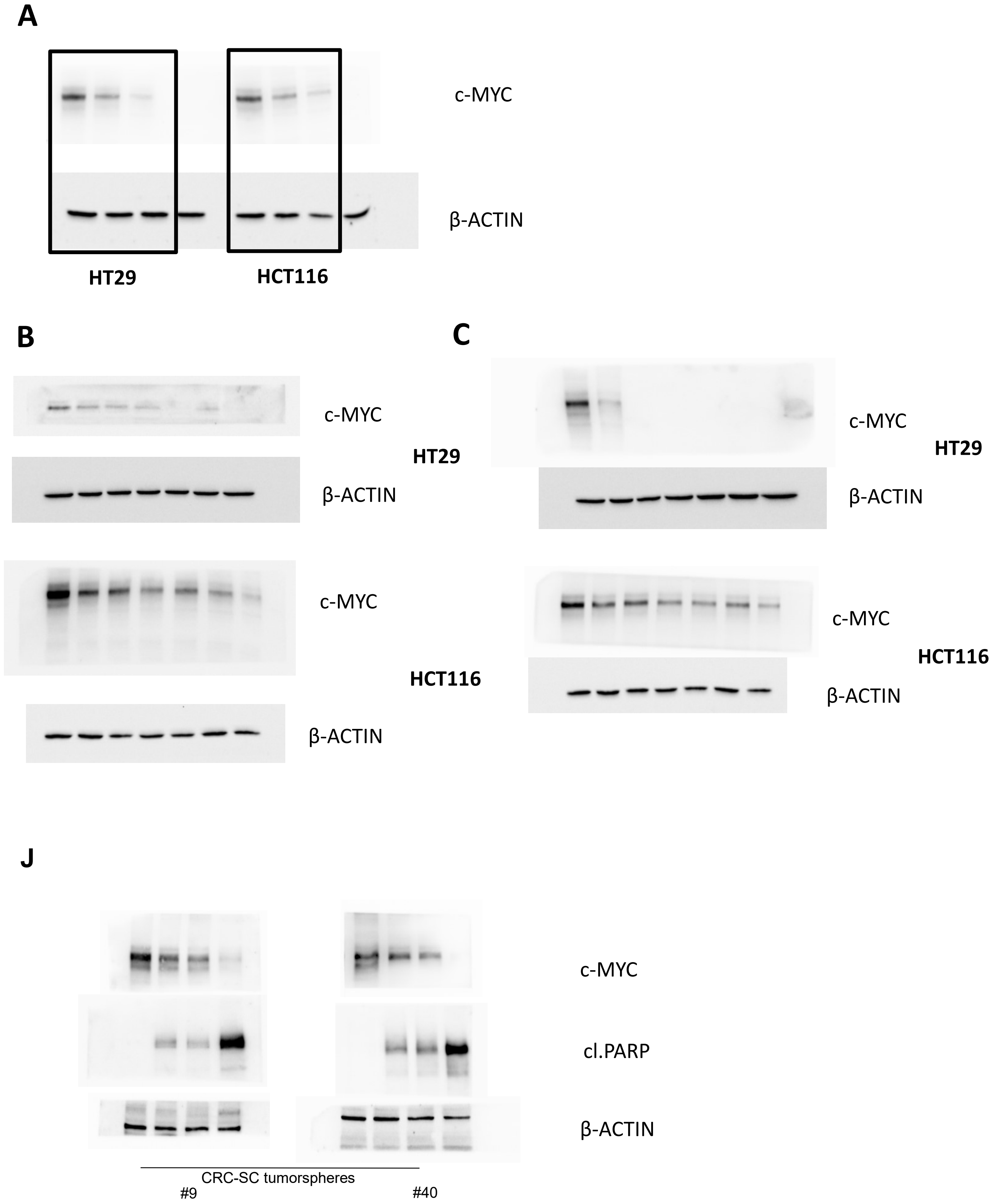

Figure 5 Original blots

A

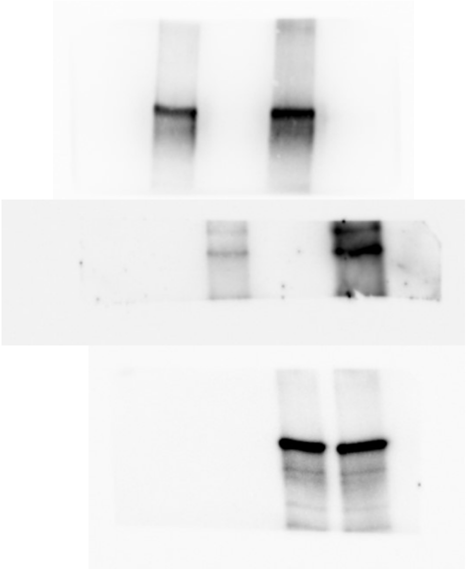

c-MYC

HIS

GST

B

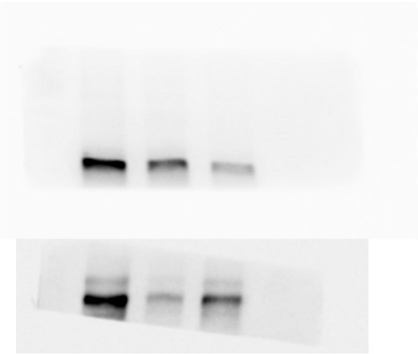

p38α

HT29

c-MYC

Supplementary Figure S1 Original blots

A

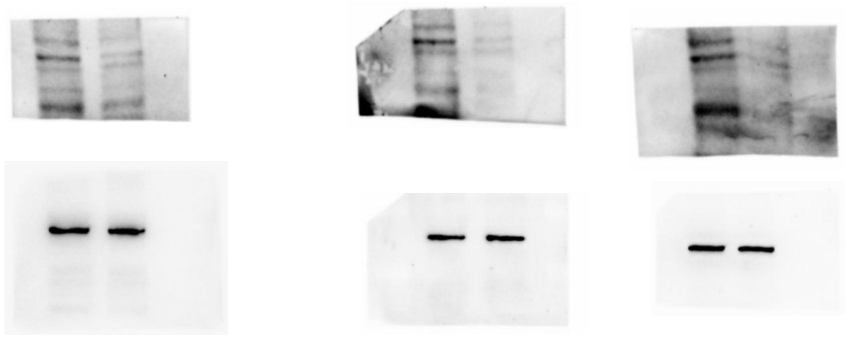

p-MK2

β-ACTIN

HT29 **HCT116** **Caco2**

B

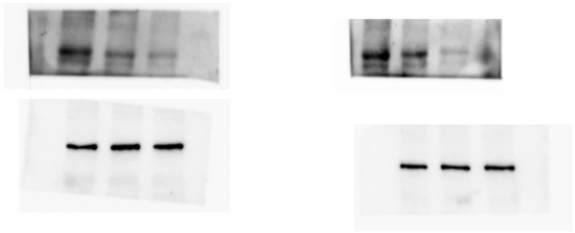

p-ERK

β-ACTIN

HT29 **HCT116**

Supplementary Figure S2 Original blots

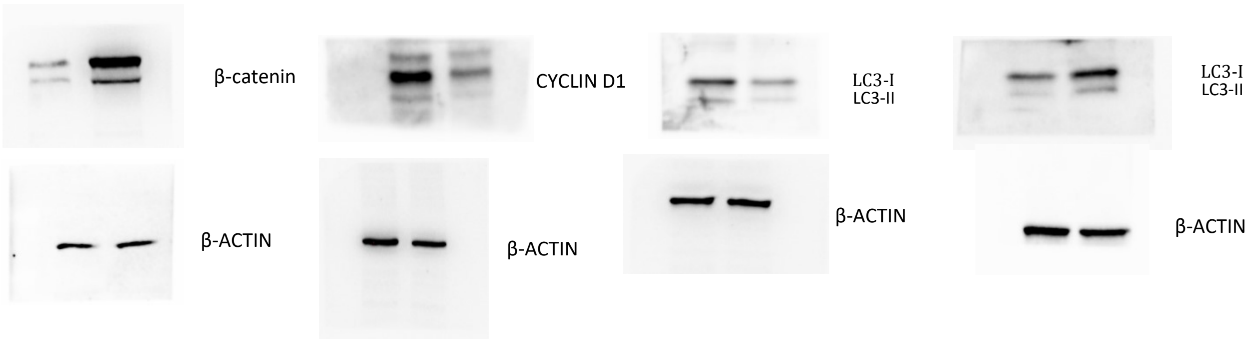

Supplement: Supplementary file 1 [file cancers-14-04840-s001.zip › cancers-1837921-supplementary.pdf]
